# Effect of Health Education on Female Teachers’ Knowledge and Practices Regarding Early Breast Cancer Detection and Screening in the Jazan Area: a Quasi-Experimental Study

**DOI:** 10.1007/s13187-018-1386-9

**Published:** 2018-06-27

**Authors:** Anwar Alameer, Mohamed Salih Mahfouz, Yahya Alamir, Nasir Ali, Abdulaziz Darraj

**Affiliations:** 1 Jazan Health Directorate, Ministry of Health, P.O. Box 1121, Abu Arish 45911, Jazan, Saudi Arabia; 2 Family and Community Medicine Department, Faculty of Medicine, Jazan University, Jazan, Saudi Arabia; 3 Health Education and Promotion Department, Faculty of Public health and Tropical Medicine, Jazan University, Jazan, Saudi Arabia

**Keywords:** Breast cancer screening, Health education, Breast self-examination, Clinical breast examination, Mammography

## Abstract

Educational programs are important tools for breast cancer prevention. The purpose of this study was to assess the effectiveness of health education in improving the knowledge and practices of female teachers regarding screening tools and the early detection of breast cancer. A two-group quasi-experimental design was conducted among 150 female teachers, who were selected from 75 schools of the Jazan General Administration of Education. Schools were chosen by a simple cluster randomization method and non-randomly assigned to either the intervention or control group. Eligible participants were recruited by a simple randomization method, proportional to the total number of teachers at each school. Those in the intervention group (*n* = 75) were compared to the control group (*n* = 75) at baseline, as well as at 6 weeks and 3 months post-intervention. Knowledge of breast cancer screening tools was measured using a modified version of the Breast Cancer Knowledge test. Breast self-examination, clinical breast examination, and mammography practices were also measured. Compared to the control group, the intervention group showed a statistically significant increase in knowledge and practice levels at both 6 weeks and 3 months post-intervention. Thus, the results of this study provide evidence that group health education programs are effective in improving breast cancer knowledge and practices in female teachers. Clinical Trial Registration number: NCT03398057.

**Electronic supplementary material:**

The online version of this article (10.1007/s13187-018-1386-9) contains supplementary material, which is available to authorized users.

## Introduction

Breast cancer (BC) is common in developed and developing countries, and it represents 23% of all cancers in women worldwide [1]. As of 2014, BC has accounted for about 28.7% of all newly diagnosed cancer in Saudi women, making it the most common cancer in this population [2]. BC incidence is expected to increase to 350% by the end of 2025, which in turn will represent a huge economic burden in the KSA [3]. Unfortunately, most BC cases in Saudi Arabia are detected in late and advanced stages with very poor prognosis. Most studies have attributed this to low awareness levels among Saudi women regarding various aspects of BC, especially early detection and screening tools [2, 4–8].

Early detection of BC is an important step to decrease the morbidity and mortality of this disease. BC screening tools include breast self-examination (BSE), clinical breast examination (CBE), and mammography. Cooperation between BC screening tools is the best way to efficiently reduce disease burden [9, 10].

Several studies of Saudi women have revealed very low levels of knowledge and usage of BC screening tools. Clearly, there is major defect in health education programs, which have so far failed to change women’s behavioral habits, even though BC screening tools have been provided free of charge [4, 5, 7, 8].

Health education is a practical, effective method to increase women’s awareness of the importance of early BC detection and its associated tools [11–16]. Group health education has proven to be effective in this field [12–14, 16], and such programs are commonly based on the health belief model (HBM) [12, 14–16]. Several studies worldwide have proven the effectiveness of HBM-based education programs in increasing BC awareness of, but few such studies have been performed in Saudi Arabia [12, 14–16]. The main objective of this study is to assess the effectiveness of a health education program in improving the knowledge and usage of screening tools for the early detection of BC, among a cohort of female teachers in Jazan, Saudi Arabia.

## Material and Methods

### Study Design

This was a quasi-experimental study carried from November 2017 to February 2018. It was registered in the Clinical Trials Registry in December 2017 (http://www.ClinicalTrials.gov).

### Participants and Setting

This study was conducted in the city of Jazan, in southwest Saudi Arabia. The targeted population included all female teachers (approximately 1400) among 75 schools belonging to the middle education office of the Jazan General Administration of Education, Ministry of Education. Teachers were chosen according to the following eligibility criteria: age ≥ 20 years, no history of BC (personally or in a first-degree relative), and not pregnant or breast feeding. Participants were excluded if they refused to give informed consent, or if any severe medical problem prevented participation. This study evaluated the effectiveness of health education in improving participants’ knowledge of BC screening tools and relevant practices, by comparing a health education group with a control group at three time points (baseline, 6 weeks, and 3 months). The dependent variables were the level of knowledge of BC detection and screening tools, and the practice thereof, including BSE, CBE, and mammography. The independent variables were the type of intervention, age, and social status.

### Sampling Procedures

Eight schools were chosen randomly according to the desired sample size (150 participants) and the average number of teachers per school (23.6). First, four schools were non-randomly allocated to the health education group, and other four were allocated to the control group. Eligible participants were recruited by a simple randomization method proportional to the number of teachers per school. The study sample size was estimated based on a similar study by Heydari et al. [14], with 60 participants in each group after an assumed 25% attrition rate after the 3-month follow-up. Accordingly, 75 participants were recruited for each group.

### Intervention

Participants in the intervention group underwent a HBM-based standardized health education program (SHEP) developed by the Heath Education Committee of the Health Education and Promotion Department, Faculty of Public Health and Tropical Medicine, Jazan University (Supplementary Material 1). SHEP included a comprehensive lecture about BC, with a deep focus on detection and screening tools, illustrated with a PowerPoint presentation containing pictures and videos. The program also included a practical BSE session. Each educational session lasted 60 min. At the end of SHEP, a focused group discussion was conducted to answer participants’ questions, and to discuss important barriers regarding BSE practice and visiting primary health care centers or clinics to undergo CBE and mammography. Several scientific and administrative solutions were discussed, with a concentration on the benefits of screening tools, both to overcome those barriers and to motivate the participants to utilize breast cancer screening tools. The interventions were conducted by three health care assistants who were trained by the Health Education Committee and the primary investigator. Participants in the control group were educated through pamphlets that included general information about BC, without any interaction with our defined measured variables. At the end of the data collection period, SHEP was presented to the control group participants and the educational material was distributed.

### Measures

The modified questionnaire used in this study comprised four parts assessing knowledge of BC screening tools and practice. The first part of the questionnaire collected sociodemographic information. The second part was a modified version of the Breast Cancer Knowledge (BCK) test developed by McCance in 1989, and assessed the participant’s knowledge of BC screening tools [17, 18]. The third part of the questionnaire assessed each participant’s BSE practices using the scale described by Champion in 1990 [19]. The fourth part of the questionnaire assessed the participant’s CBE and mammography practices, based on questionnaires by de Oliveira et al. and Wang J.H. et al. [15, 20]. In total, the questionnaire included 38 items, with a mix of multiple-choice and yes/no questions in addition to demographic data. Each part of the instrument has been rigorously tested for reliability and validity (see Supplementary Material 2).

### Data Collection Procedure

Data were collected using the same self-administered questionnaire at the three time points. On average, it took 20 min to finish the questionnaire. Data for the first time point was collected from all participants within 4 weeks. The 6-week data was collected within 2 weeks with a 100% response rate in both groups. The 3-month data was also collected within 2 weeks, with response rates of 98.7 and 97.3% in the intervention and control groups, respectively. The losses to follow up were due to either the inability to make contact or refusal to participate.

### Statistical Analysis

Data were analyzed using the Statistical Package for the Social Sciences, version 23 (SPSS). Descriptive statistics, the independent *t* test, the chi-squared test, the Mann–Whitney *U* test, the Wilcoxon signed-rank test, McNemar’s test, the Friedman test, and logistic regression were used for data analysis.

## Results

### Demographic Information

The participants in the intervention and the control groups had similar demographic characteristics, with no statistically significant differences (Table 1).

**Table 1:** Sociodemographic characteristics in the intervention and control groups at baseline

Demographic		Intervention group			Control group			Statistic	*P*
			*n*	%		*n*	%		
Age (M ± SD)		39.03 ± 4.96	75		38.97 ± 4.43	75		0.069^*^	0.945
< 40		35.3 ± 3.1	39	52	35.8 ± 2.8	41	54.7		
≥ 40		43.3 ± 3.1	36	48	42.7 ± 2.70	34	45.3		
Marital status	Single		10	13.3		10	13.3	1.444^**^	0.780
	Married		60	80		63	84		
	Widowed		3	4		1	1.3		
	Divorced		2	2.7		1	1.3		

*M*, mean; *SD*, standard deviation. *Independent t test; **Fisher’s exact test

### Breast Cancer Knowledge Test

Most of the participants (*n* = 142, 94.7%) stated that mammography can detect unfelt lumps easily, and 89.3% (*n* = 134) viewed BSE as effective for detecting breast cancer. In contrast, a minority of the participants (28%, *n* = 42) realized the importance of practicing BSE in conjunction with undergoing CBE and mammography. Only 20.7% (*n* = 31) knew the recommended age to undergo mammography as recommended by Saudi Arabian guidelines. On the other hand, 85.3% (*n* = 128) knew about the increasing risk of BC with age. Only 26.7 and 22.7% of participants in the intervention and control groups, respectively, were aware of abnormalities in nipple discharge. In contrast, 60% of participants in both groups understood the symptoms of BC.

The differences between participants in the intervention and control groups at baseline were not statistically significant for any knowledge items related to BC detection and screening tools. The overall knowledge scores of both groups before intervention were similar (*P* = 0.419). In contrast, the knowledge scores significantly differed between the groups, both 6 weeks and 3 months after intervention (*P* < 0.001) (Table 2). The knowledge scores were increased in the intervention group post intervention (*P* < 0.001, odds ratio [OR] = 29.5, 95% confidence interval [CI] = 11.49–75.70). The pre- and post-intervention knowledge scores in the control group were not significantly different (*P* = 0.692) (Table 2).

**Table 2:** Between- and within-group comparisons of knowledge and practice scores for breast cancer screening tools for the intervention (IG) and control groups (CG)

Item	Score			
	Time	Intervention group, median ± IQR	Control group, median ± IQR	*P* ^*^
Overall knowledge score^1^	Before intervention	9 (3)	10 (5)	*P* = 0.419
	6 weeks after intervention	15 (3)	9.5 (5)	*P* < 0.001
	3 months after intervention	15 (2.5)	9.5 (5)	*P* < 0.001
	*P* ^**^	*P* < 0.001	*P* = 0.747	
BSE score^2^	Before intervention	18 (8.25)	17 (7)	*P* = 0.722
	6 weeks after intervention	38.5 (9.25)	18 (12)	*P* < 0.001
	3 months after intervention	37.5 (8.25)	17 (14)	*P* < 0.001
	*P* ^**^	*P* < 0.001	*P* < 0.001	

Overall knowledge score was calculated by summing the number of correct responses, with possible scores ranging from 0 to 19. Each correct response was worth one point. *BSE*, breast self-examination. The BSE score was calculated by summing the number of correct responses, with possible values ranging from 0 to 45. Each completely correct response was worth three points

Statistical tests: *Mann–Whitney *U* test (between IG and CG pre-intervention, 6 weeks and 3 months post-intervention), **Freidman test (within-groups comparison). *IQR*, interquartile range

### Breast Self-Examination Scale

Of the entire study sample, 57.3% (*n* = 86) practiced BSE before health education, and the BSE scores of the two groups were similar (*P* = 0.722). On the other hand, the BSE scores were significantly different between groups 6 weeks and 3 months after intervention (*P* < 0.001). Three months after health education, 93.2% (*n* = 69) of the intervention group and 58.9% (*n* = 43) of the control group practiced BSE (*P* < 0.001). Overall, the BSE score was significantly increased in the intervention group (*P* < 0.001, OR = 26.25, 95% CI = 8.26–83.42) (Tables 2 and 3).

**Table 3:** Pre- and post-intervention comparison of practices regarding breast cancer screening tools between female teachers in the intervention (IG) and control groups (CG)

Practice item	Pre-intervention			6 weeks post-intervention			3 months post-intervention		
	IG	CG	Significance	IG	CG	Significance	IG	CG	Significance
	(*N* = 75)	(*N* = 75)		(*N* = 75)	(*N* = 75)		(*N* = 74)	(*N* = 73)	
Practicing BSE^*^									
Yes	43 (57.3%)	43 (57.3%)	*χ*^2^ = 0.000	69 (92%)	43 (57.3%)	*χ*^2=^23.825	69 (93.2%)	43 (58.9%)	*χ*^2=^23.887
No	32 (42.7%)	32 (42.7%)	*P* = 1.000	6 (8%)	32 (42.7%)	*P* < 0.001	5 (6.8%)	30 (41.1%)	*P* < 0.001
Underwent CBE^**^ without feeling anything									
Yes	14 (18.7%)	15 (20.0%)	*χ*^2^ = 0.043	53 (70.7%)	16 (21.3%)	*χ*^2^ = 36.742	55 (74.3%)	16 (21.9%)	*χ*^2^ = 40.418
No	61 (81.3%)	60 (80.0%)	*P* = 0.836	22 (29.3%)	59 (78.7%)	*P* < 0.001	19 (25.7%)	57 (78.1%)	*P* < 0.001
Underwent an annual CBE^**^									
Yes	14 (18.7%)	13 (17.3%)	*χ*^2^ = 0.045	53 (70.7%)	14 (18.7%)	*χ*^2^ = 41.027	55 (74.3%)	15 (20.5%)	*χ*^2^ = 42.606
No	61 (81.3%)	62 (82.7%)	*P* = 0.832	22 (29.3%)	61 (81.3%)	*P* < 0.001	19 (25.7%)	58 (79.5%)	*P* < 0.001

*Breast self-examination, **Clinical breast examination, *χ*^2^ Chi-square test

### Clinical Breast Exam and Mammography

Compared to the control group, there was a significant increase in the level of CBE and mammography practice among the teachers in the intervention group at 6 weeks and 3 months after health education program (*P* < 0.001 and *P* = 0.001, respectively; Tables 3 and 4).

**Table 4:** Pre- and post-intervention comparison of BC screening tools between female teachers in the intervention (IG) and control groups (CG)

Practice Items	Pre-intervention			6 weeks post-intervention			3 months post-intervention		
	IG	CG	Significance	IG	CG	Significance	IG	CG	Significance
	(*N* = 36)	(*N* = 34)		(*N* = 36)	(*N* = 34)		(*N* = 36)	(*N* = 32)	
Underwent a mammogram, for women ≥ 40 years									
Yes	14 (38.9%)	11 (32.4%)	*χ*^2^ = 0.325	25 (69.4%)	11 (32.4%)	*χ*^2^ = 9.630	27 (75.0%)	11 (34.4%)	*χ*^2^ = 11.341
No	22 (61.1%)	23 (67.6%)	*P* = 0.568	11 (30.6%)	23 (67.6%)	*P* = 0.002	9 (25.0%)	21 (65.6%)	*P* = 0.001
Last mammogram									
Less than 1 year	1 (7.1%)	2 (18.2%)	*χ*^2*^ = 2.083 *P* = 0.603	17 (68.0%)	2 (18.2%)	*χ*^2*^ = 13.147 *P* = 0.003	21 (77.8%)	3 (27.3%)	*χ*^2*^ = 12.574 *P* = 0.003

*χ*^2^ Chi-square test, *χ*^2*^ Fisher’s exact test

## Discussion

This is one of the few studies in Saudi Arabia to assess the benefits of a group health education program on improving female teachers’ knowledge and utilization of screening tools for the early detection of breast cancer.

Group health education has proven to be effective in increasing the knowledge and practice of breast cancer screening tools in different populations [12–14, 16]. The results of the present study imply that our group health education program was effective in improving knowledge and breast cancer screening practices among female teachers in the Jazan area. These results are consistent with those of previous studies and support the role of HBM-guided interventions in this area [12, 14–16].

Improved knowledge levels had a positive impact on behavioral change by increasing self-confidence among targeted women. High confidence levels lead to improved usage of BC screening tools and ensure the sustained value of screening tools, especially with close monitoring of participants, as previously demonstrated by a study in Sri Lanka [12, 13].

The present study’s results demonstrated the effectiveness of group health education on increasing BSE practices among female teachers, especially with its comprehensive practical session using a silicone breast model to simulate how to discover a lump and to enable a deep understanding of abnormal breast changes [12]. Focused group discussion has been demonstrated to change women’s health beliefs regarding the benefits of BC screening tools, and to motivate them to apply these tools, as shown in previous studies of populations in Malaysia and Turkey [12, 16].

We also confirmed the effectiveness of health education based on HBM in overcoming several barriers mentioned in previous studies of Saudi women. Of the study participants, 74.3% underwent CBE after the group health education program, compared to 18.7% before it. Moreover, 75% of the subjects ≥ 40 years of age underwent mammography after the group health education program, compared with 38.9% before. These results regarding mammograms are consistent with those of a similar study in Iran [14].

Finally, although group health education is more cost-effective and less time-consuming than individual health education methods [11, 14], evidence suggests that group and individual health education programs are equally effective [21]. In contrast, multimedia health education is an even more time- and cost-effective method than group health education. While group health education was more effective than multimedia health education in improving knowledge and practice levels of mammography among female teachers in an Iranian study, multimedia programs have the advantages of simplicity, flexibility, and reproducibility [14]. The current study only demonstrated the effectiveness of group health education in achieving short-term outcomes. Intermediate and long-term outcomes must be tested in future research by using longer follow-up periods. Moreover, logical program models need to be applied to optimize the achievement of intermediate and long-term outcomes among targeted populations, requiring strong governmental support to have a significant effect at the population level [22].

### Limitations

The short follow-up period and lack of close monitoring were important limitations of this study. Longer follow-up periods can enable more women to undergo CBE and mammography and would provide enough time to test the sustainability of BSE practices. It could also provide an excellent chance to evaluate intermediate and long-term outcomes among women. Moreover, close monitoring would allow for the effectiveness of this health education program to be more precisely evaluated in the long term. Finally, the current study was conducted only in female teachers, which is an important limitation that reduces the generalizability of the results to the general population in Saudi Arabia.

## Conclusion

Our group health education program, based on HBM, was effective in improving knowledge and practices regarding BC screening tools among female teachers in Jazan area schools. There is great opportunity for researchers in Saudi Arabia to study a variety of health education methods, behavioral change models, and various targeted populations in this field. Moreover, qualitative research should be performed to gain an in-depth understanding of the perception, awareness, and attitudes regarding breast cancer screening tools in target populations. Such findings would be valuable for exploring the various barriers hindering the use of breast cancer screening tools at the community level.

## Electronic supplementary material

ESM 1 (DOCX 21 kb)

## Abbreviations

BCK: breast cancer knowledge
BSE: breast self-examination
CBE: clinical breast examination
BC: breast cancer
HBM: health belief model
SHEP: standardized health education program
