# Supplementary material for: Effect of Health Education on Female Teachers’ Knowledge and Practices Regarding Early Breast Cancer Detection and Screening in the Jazan Area: a Quasi-Experimental Study
Source: J Cancer Educ. 2018 Jun 27;34(5):865–70. doi: 10.1007/s13187-018-1386-9 (PMC6785574; doi:10.1007/s13187-018-1386-9)
Supplement: Supplementary file 1 — (DOCX 21 kb) [file 13187_2018_1386_MOESM1_ESM.docx]

**Supplementary Material 1**

Summary of the standardized health education program (SHEP)

| **Unit** | **Content** |
| --- | --- |
| **Overview** | - Introduction to the content of the booklet - Overall learning objectives - Target population |
| **Unit 1** | *Breast Cancer (BC) awareness:*   - What is BC? - Symptoms and signs of BC - Risk factors of BC - Stages of BC - Treatment of BC |
| **Unit 2** | *Prevention methods:* knowledge and practice regarding early detection and screening tools of BC and their importance.   - Breast self-examination (BSE) - Clinical breast examination (CBE) - Mammography |

**Supplementary Material 2**

Assessment Battery

| Scale | Items | Variables | Reliability | Population |
| --- | --- | --- | --- | --- |
| Breast Cancer Knowledge (BCK) test | 19 | *Knowledge regarding breast cancer screening tools  *General breast cancer information | Internal Consistency (Cronbach alpha=0.745) | Female teachers |
| Breast self-examination (BSE) scale | 15 | *Knowledge and practice regarding BSE. | Internal Consistency (Cronbach alpha ranging from 0.653-0.721) | Female teachers |
| Practice regarding clinical breast examination (CBE) and mammography | 4 | * Practice regarding CBE and mammography. | Internal Consistency (Cronbach alpha=0.865) | Female teachers |

**Supplementary Material 3**

**Scoring**

The scoring system was developed to assess the knowledge and practice of breast cancer screening tools in a target sample with expert participation. Knowledge scores were taken from Guilford et al. (2011). The overall knowledge score was calculated by counting the number of correct responses, with possible scores ranging from 0-19. Each correct response was worth one point. An absolute cutoff point of 60% was used to detect improved knowledge or the absence thereof. A participant score of 0-11 demonstrated poor knowledge, while a score of 12-19 demonstrated good knowledge.

The breast self-examination (BSE) scoring system was developed by the primary investigator with expert participation. BSE scores were calculated by summing the number of correct responses, with possible values ranging from 0-45. Each correct response was worth three points. Sample items were as follows: “Have you ever done breast self-examination?” and “when doing a breast self-examination, how many fingers do you use?” Other questions were worth 1-3 points, depending on the quality of correct responses. These items were as follows: “How often do you lie on your side when examining the outside area of your breast?” and “when examining your breasts, how often do you move your fingers in small dime-shaped circles?” For the first of these graded questions, scores were given as follows: “never lie on side” (0 points), “sometimes lie on side” (1 point), “frequently lie on side” (2 points), “always lie on side” (3 points). For the second graded question, the values were: “never use small circles” (0 points), “sometimes use small circles” (1 point), “frequently use small circles” (2 points), “always use small circles” (3 points). An absolute cutoff point of 60% was used to detect improved BSE practice. Scores of 0-26 demonstrated poor practice, while scores of 27-45 demonstrated good practice.
